# Role of PCSK5 Expression in Mouse Ovarian Follicle Development: Identification of the Inhibin α- and β-Subunits as Candidate Substrates

**DOI:** 10.1371/journal.pone.0017348

**Published:** 2011-03-08

**Authors:** Monica Antenos, Lei Lei, Min Xu, Anjali Malipatil, Sarah Kiesewetter, Teresa K. Woodruff

**Affiliations:** 1 Department of Obstetrics and Gynecology, Feinberg School of Medicine, Northwestern University, Chicago, Illinois, United States of America; 2 Center for Reproductive Science, Northwestern University, Evanston, Illinois, United States of America; 3 Robert H. Lurie Comprehensive Cancer Center of Northwestern University, Chicago, Illinois, United States of America; State Key Laboratory of Reproductive Biology, Institute of Zoology, Chinese Academy of Sciences, China

## Abstract

Inhibin and activin are essential dimeric glycoproteins belonging to the transforming growth factor-beta (TGFβ) superfamily. Inhibin is a heterodimer of α- and β-subunits, whereas activin is a homodimer of β-subunits. Production of inhibin is regulated during the reproductive cycle and requires the processing of pro-ligands to produce mature hormone. Furin is a subtilisin-like proprotein convertase (proconvertase) that activates precursor proteins by cleavage at basic sites during their transit through the secretory pathway and/or at the cell surface. We hypothesized that furin-like proconvertases are central regulators of inhibin α- and β-subunit processing within the ovary. We analyzed the expression of the proconvertases furin, PCSK5, PCSK6, and PCSK7 in the developing mouse ovary by real-time quantitative RT-PCR. The data showed that proconvertase enzymes are temporally expressed in ovarian cells. With the transition from two-layer secondary to pre-antral follicle, only PCSK5 mRNA was significantly elevated. Activin A selectively enhanced expression of PCSK5 mRNA and decreased expression of furin and PCSK6 in cultured two-layer secondary follicles. Inhibition of proconvertase enzyme activity by dec-RVKR-chloromethylketone (CMK), a highly specific and potent competitive inhibitor of subtilisin-like proconvertases, significantly impeded both inhibin α- and β-subunit maturation in murine granulosa cells. Overexpression of PC5/6 in furin-deficient cells led to increased inhibin α- and β_B_-subunit maturation. Our data support the role of proconvertase PCSK5 in the processing of ovarian inhibin subunits during folliculogenesis and suggest that this enzyme may be an important regulator of inhibin and activin bioavailability.

## Introduction

Growth and development of primordial follicles to ovulatory status is associated with marked proliferation, recruitment and differentiation of somatic cells and changes in oocyte size and morphology that reflect both nuclear and cytoplasmic maturation. Inhibins, activins and follistatins were first identified in ovarian follicular fluid by their ability to modulate the secretion of FSH from pituitary gonadotrophs. Inhibins and follistatin suppress FSH secretion, whereas activins enhance FSH secretion. Although a long-loop negative feedback role for ovarian inhibins in the regulation of FSH secretion is now well established in both sexes [1], [2], [3], it is unlikely that activins of ovarian origin exert endocrine effects on the pituitary. Rather, activins are produced by, and subserve local regulatory roles in a diverse range of tissues, including the anterior pituitary. Recently, we identified a positive short feedback loop in pituitary gonadotrope cells that augments the secretion of bioactive mature activin B and inhibin B necessary for local FSH regulation [4]. The granulosa cell of the ovary produces inhibin A and inhibin B; however, the secretion patterns for these ligands are distinct, suggesting an underlying control over biosynthesis and release. Preliminary studies in our lab described differential secretion patterns of inhibin A and inhibin B during the rat reproductive cycle, differential compartmentalization of inhibin subunit protein in granulosa cells of developing follicles, and the differential regulation of biosynthetic processing of inhibin isoforms [5]. Like TGFβ and many other growth factors, the inhibin α-, β_A_- and β_B_-subunits are cleaved by proconvertases to generate mature active peptides. Proconvertases are a family of serine proteases structurally related to the endoproteases bacterial subtilisin and yeast kexin, and cleave precursors of polypeptides at the carboxylterminal end of single or paired basic residues. To date, seven mammalian proconvertases have been identified [6], [7], [8], [9], [10], [11], [12]. Of these, furin (PCSK3), PC5/6 (PCSK5), PACE4 (PCSK6) and PC7 (PCSK7) exhibit widespread tissue distribution, whereas PC1 (PCSK1) and PC2 (PCSK2) are expressed only in endocrine and neuroendocrine tissues and cells [12], [13], [14]. PC5/6 is encoded as two splice variants: PC5/6A and PC5/6B [7], [8], [15]. PC5/6A is the major isoform in most tissues, whereas PC5/6B is predominately expressed in the intestine and kidney [16].

An early step in the biosynthesis of inhibin involves the attachment of N-linked oligosaccharides to the inhibin subunits. We have demonstrated that specific oligosaccharide attachment to the α- and β_A_-subunits direct the differential assembly and secretion of inhibin A and activin A dimers [17]. We further showed that the inhibin α- and β_B_-subunits are targets of furin processing and that activin A positively regulates furin through a Smad2/3-dependent pathway in pituitary gonadotrope cells [4]. Here, we extend these studies by characterizing the mRNA expression of the α-, β_A_- and β_B_-subunits and the proconvertases (furin, PCSK5, PCSK6, and PCSK7) during early mouse ovarian development and folliculogenesis. We found that inhibin bioactivity and maturation in the ovary involves the activation of different proconvertases, and we provide novel evidence that the inhibin α- and β_B_-subunits are substrates of the proconvertase PC5/6 and that activin A is a likely regulator of this enzyme in mouse granulosa cells. These studies provide insight into the control of inhibin and activin synthesis in mouse ovarian granulosa cells and contribute to a more complete understanding of the processes involved in normal folliculogenesis, as well as the potential mechanisms that may underlie infertility secondary to inappropriate ovarian hormone production and secretion.

## Materials and Methods

### Recombinant ligands, plasmids and chemical reagents

Recombinant human activin A was purified as reported previously [18]. Recombinant activin B (R&D Systems, Minneapolis, MN) was reconstituted in 0.1% BSA in phosphate buffered saline (PBS). The human inhibin α-subunit and β_B_-subunit cDNAs were provided by Genentech (South San Francisco, CA). Human furin, PC5/6A, PC5/6B and PC7 expression plasmids were a kind gift from Dr. J.W. Creemers (Katholieke Universiteit Leuven, Belgium). The Pace4 expression plasmid was a kind gift from Dr. A. Tsuji (University of Tokushima, Japan). The furin inhibitor, dec-RVKR-chloromethylketone (CMK) was purchased from Alexis Biochemicals (San Diego, CA). Site-directed mutagenesis of the α-subunit cleavage site was performed by PCR as described previously [17]. Primers used for mutagenesis of the inhibin α-subunit cleavage site are: RARR→RARA (forward: ccacccagtggaggggagaga *gcc*cga*gcc*tcaactcccctg; reverse: caggggagttga*ggc*tcg*ggc*tctctcccctccactgggtgg) and RARR→RAAA (forward: ccacccagtggaggggagaga*gccgccgcc*tcaactcccctg; reverse: caggggagttga*ggcggcggc*tctctcccctccact gggtgg). Mutants were confirmed by DNA sequence analysis at the Northwestern University Biotech Core Facility.

### Animals

Ovaries were removed from prepubertal 2-, 6-, 10- and 19-day-old female F1 hybrids (C57BL/6XCBA). Primary cultures of mouse granulosa cells were isolated from adult mice by needle puncture as previously described [19]. Breeding pairs and prepubertal mice were housed in a temperature-controlled and light-controlled environment (14L∶10D), and were provided with food and water ad libidum. Animals were treated in accordance with the National Institutes of Health Guide for the Care and Use of Laboratory Animals. The protocols used were approved by the IACUC (ACUC protocol 2003-0026) at Northwestern University. Unless otherwise noted, all media formulations were purchased from Invitrogen (Carlsbad, CA).

### Cell lines

The GRMO2 mouse granulosa cell line was provided by N.V. Innogenetics (Ghent, Belgium) [20]. GRMO2 cells were grown in HDTIS (1∶1 mixture of DMEM and Ham's F-12 medium, 10 µg/ml insulin, 5 nM sodium selenite and 5 µg/ml transferrin supplemented with 2% fetal bovine serum and sodium pyruvate [100 mg/liter]) in a humidified incubator at 37°C and 5% CO_2_ as described previously [20]. A furin-deficient human colon adenocarcinoma cell line (LoVo) was obtained from the ATCC (Rockville, MD). LoVo and human embryonic kidney (HEK 293) cells were grown and maintained in DMEM supplemented with 10% fetal calf serum and 1% antibiotic as described [4].

### Follicle isolation, encapsulation and culture

Primary, two-layer secondary, multi-layer secondary and antral follicles were mechanically isolated and collected from day 8, 12, 16, 21–22 mice as described below. Follicles were classified as follows: primordial follicles (<50 µm); primary follicles (50–100 µm; harvested only from day 8 mouse ovaries); two-layer secondary follicles (100–130 µm; harvested only from day 12 mouse ovaries); multilayer secondary follicles (150–180 µm; harvested only from day 16 mouse ovaries); antral follicles (280–340 µm; harvested only from days 21–22 mouse ovaries) and preovulatory follicles (>500 µm; harvested only from >day 22 ovaries) (20–22). Follicles were flash frozen in liquid nitrogen prior to RNA isolation. For RNA isolation, follicles were pooled by size: 110 primary follicles, 45 secondary follicles; 25 multilayer secondary follicles; 15 early antral follicles and 12 pre-ovulatory follicles. RNA was purified using the Absolutely RNA Microprep Kit (Stratagene, Cedar Creek, TX) according to the manufacturer's protocol. RNA quality was verified using the RNA 6000 Nano Chip Assay (Agilent Technologies, Santa Clara, CA).

For follicle culture experiments, two-layer secondary follicles (100–130 µm diameter) were mechanically isolated from the ovaries of 12-day-old females and encapsulated in alginate as previously described [21], [22], [23]. Throughout isolation, encapsulation and culture, the follicles were maintained at 37°C and pH 7. After a series of experiments to determine effective activin A and activin B concentrations on inhibin α- and β_B_-subunits in immortalized cell lines (data not shown; Antenos et al., 2008), follicles were cultured for a 4-day period in culture media consisting of αMEM, 3 mg/ml BSA, 10 mIU/ml rFSH, 5 µg/ml insulin, 5 µg/ml transferrin and 5 ng/ml selenium supplemented with 50 ng/ml of activin A or activin B. The concentration of activin used is similar to the doses of activin known to promote human and ovine follicle development [24], [25]. Each encapsulated follicle occupied a single well of a 96-well plate. After 2 days of culture, the media in each well was changed and fresh culture media supplemented with vehicle, activin A or activin B was added. Follicle diameter and overall health was assessed using a Leica DM IRB microscope with transmitted light and phase objectives (Leica, Bannockburn, IL). At the end of the 4-day culture, follicles were removed from alginate beads using alginate lysase (10 IU/ml). Follicles from each condition were collected, pooled (16–20/group), and subjected to real-time RT-PCR analysis as described below.

### Quantitative real-time RT-PCR

RNA was reverse transcribed into first strand cDNA using the Advantage Reverse PCR kit (Clontech, Mountain View, CA). Real-time RT-PCR was programmed according to the manufacturer's instructions. From the original RT reaction, 1.5 µl was subjected to PCR amplification in a 25 µl volume with Taqman Universal PCR Master Mix (Applied Biosystems, Foster City, CA) under the following conditions: 50°C hold for 2 min, 95°C hold for 10 min, then 40 cycles of 95°C for 15 s, and 60°C for 1 min. Primer and probe sets were FAM (6-carboxy-fluorescein) labeled and acquired from Applied Biosystems Assays on demand and designed to span intron/exon borders for inhibin β_A_ (Mm00434338), β_B_ (Mm01286587), α (Mm00439683), furin (Mm00440646), PCSK5 (Mm01206139), PCSK6 (Mm01319134) and PCSK7 (Mm00476614). Data were normalized with VIC-labeled GAPDH as the internal control (ΔC_t_). The threshold levels of GAPDH were not altered by any treatments. Standard error from the mean from replicates are represented as 2∧−ΔΔC_t_ as described [26].

### Immunoblot analysis

Ovaries were homogenized in lysis buffer supplemented with Complete Protease Inhibitor Tablets (Roche, Madison, WI) for immunoblotting procedures as described [17]. Goat anti-rabbit horseradish peroxidase-conjugated secondary antibody was purchased from Zymed Laboratories. Immunoblot results were visualized using an ECL detection reagent (Amersham Biosciences, Inc., Buckinghamshire, England) and exposed at varying time points onto an X-ray film (Kodak, Rochester, New York, USA).

### Proconvertase inhibition

The furin inhibitor, dec-RVKR-CMK, was added to cultures of GRM02 cells for 48 h. Media was collected, trichloroacetic acid (TCA) precipitated and subjected to immunoblot analysis under reducing conditions.

### Transfection

To assess processing of the inhibin subunits in HEK293 and LoVo cells, cells were plated in a 24-well plate 1 day prior to transfection at a confluency of ∼80%. Cells were transfected with empty vector, the wild type or cleavage site mutant inhibin α-subunit expression plasmids or the proconvertase expression plasmids as recommended by manufacturer and total DNA transfected did not exceed 0.8 µg/well. After 18 h, media was collected, (TCA) precipitated and analyzed by immunoblotting procedures under reducing conditions.

*Statistics:* Values are reported as means ± SEM and were analyzed using Prism (Version 4.0a) (GraphPad Software, Inc., San Diego, CA). Analysis of variance (ANOVA), followed by Tukey's or Bonferonni post-tests, was used to evaluate differences between treatments. Statistical significance was reported if p<0.05.

## Results

### Expression of inhibin subunit mRNA transcripts and protein in mouse ovaries

**Figure 1:**
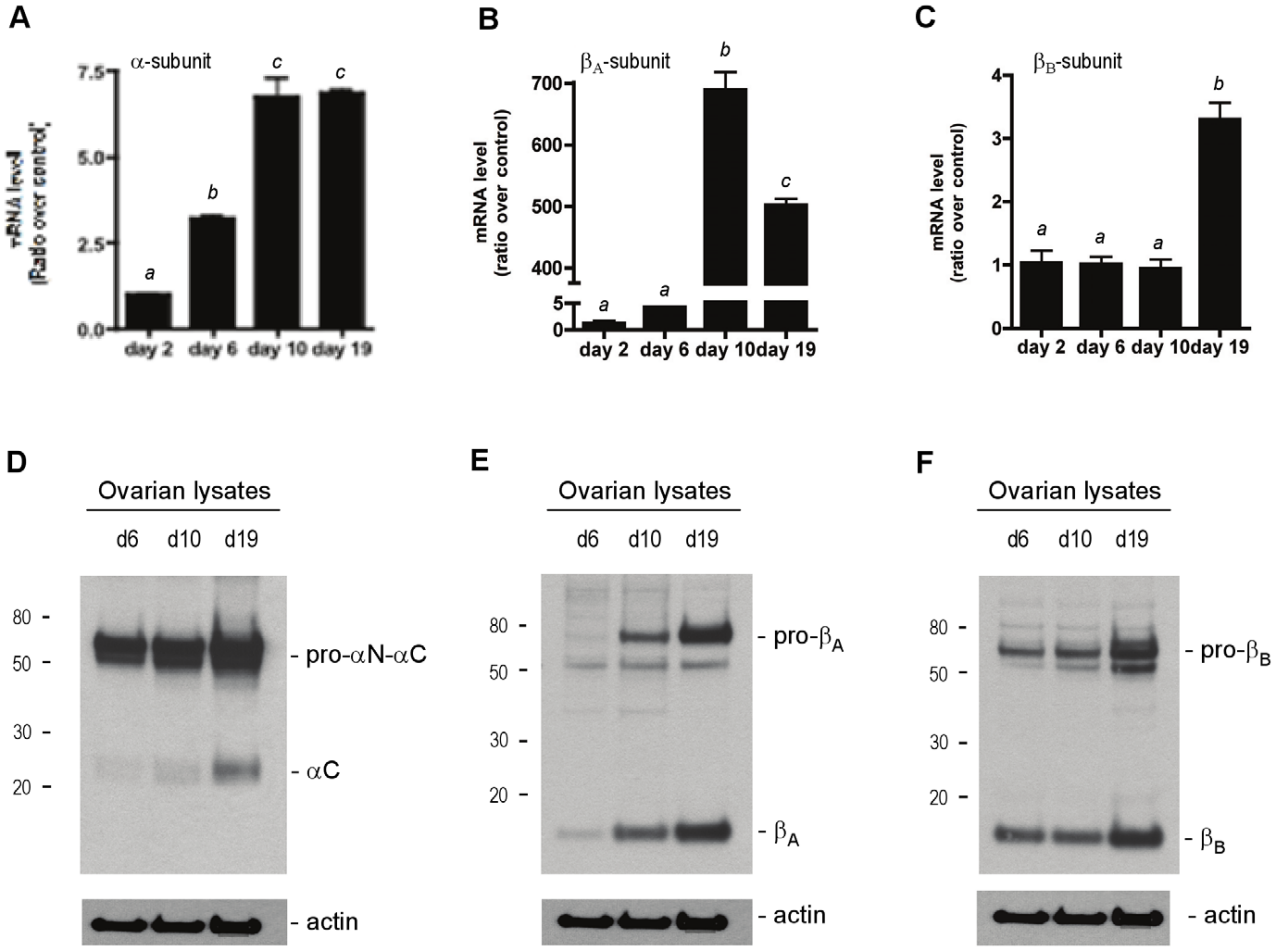
Expression of inhibin α-, β_A_- and β_B_-subunits in the postnatal mouse ovary. Basal expression levels of inhibin α- (A), β_A_- (B) and β_B_- (C) subunits in developing mouse ovaries were determined by real-time RT-PCR. The mRNA levels of the subunits are shown as a ratio over the day 2 ovary mRNA levels (control). Detection of the inhibin α- (D), β_A_- (E) and β_B_- (F) subunit protein in ovarian lysates from day 6, day 10 and day 19 ovaries. Ovarian lysates were collected, separated under reducing conditions; each subunit was detected by immunoblotting with the corresponding rabbit polyclonal antibody. Forty µg of protein was loaded per lane. Equal loading of lysates was confirmed with an anti-actin antibody.

Numerous experiments to date have demonstrated that the inhibin α-, β_A_- and β_B_-subunits are produced in granulosa cells of the ovary and have very limited expression in other ovarian cells, such as the epithelial, stromal or theca cells. We were interested in determining the expression of these subunits and the activation of these proteins as follicles grow. We first wanted to determine the expression profiles of the inhibin subunits in day 2, 6, 10 and 19 whole mouse ovaries by real-time RT-PCR. Ovaries from mice of specific ages (d2, d6, d10 and d19) were pooled and mRNA transcript levels of the inhibin α-, βA- and βB-subunits were assessed. A significant 3-fold change in the inhibin α-subunit mRNA levels was observed between day 2 and day 6 (Figure 1A). The mRNA levels for this subunit continued to increase to approximately 7-fold at day 10, and at day 19. A 4-fold increase in β_A_-subunit was observed at day 6 compared with day 2 ovaries (Figure 1B), followed by a vast increase in β_A_-subunit levels by day 10 of ovary development. These levels decreased by day 19; however, abundant levels of the inhibin β_A_-subunit remained. In contrast to the β_A_-subunit, the levels of the β_B_-subunit transcript varied little between day 2, 6 and 10 ovaries (Figure 1C), but by day 19, the levels of the β_B_-subunit mRNA had increased approximately 3-fold.

We next examined the levels of the inhibin subunits in ovarian protein lysates (40 µg/lane) from day 6, 10 and 19 ovaries by immunoblot. The relative amounts of each subunits protein increased with ovarian development, and as more protein was observed, a concomitant increase in the relative amounts of processed or cleaved mature subunits accumulated (Figures 1D–F). Day 19 ovarian lysates contained the highest levels of inhibin subunit proteins and also contained the most processed mature forms in the ovarian lysates. The relative increase in subunit expression closely matched those observed at the mRNA level and confirmed that the inhibin subunit genes and proteins are expressed and dynamically up-regulated during ovarian follicle development. The dynamic regulation of the inhibin subunits led us to investigate the expression and regulation of proconvertases thought to be necessary for maturation of inhibin and activin peptide hormones in ovarian granulosa cells.

### Furin, PCSK5, PCKS6 and PCSK7 mRNA expression in the mouse ovary

**Figure 2:**
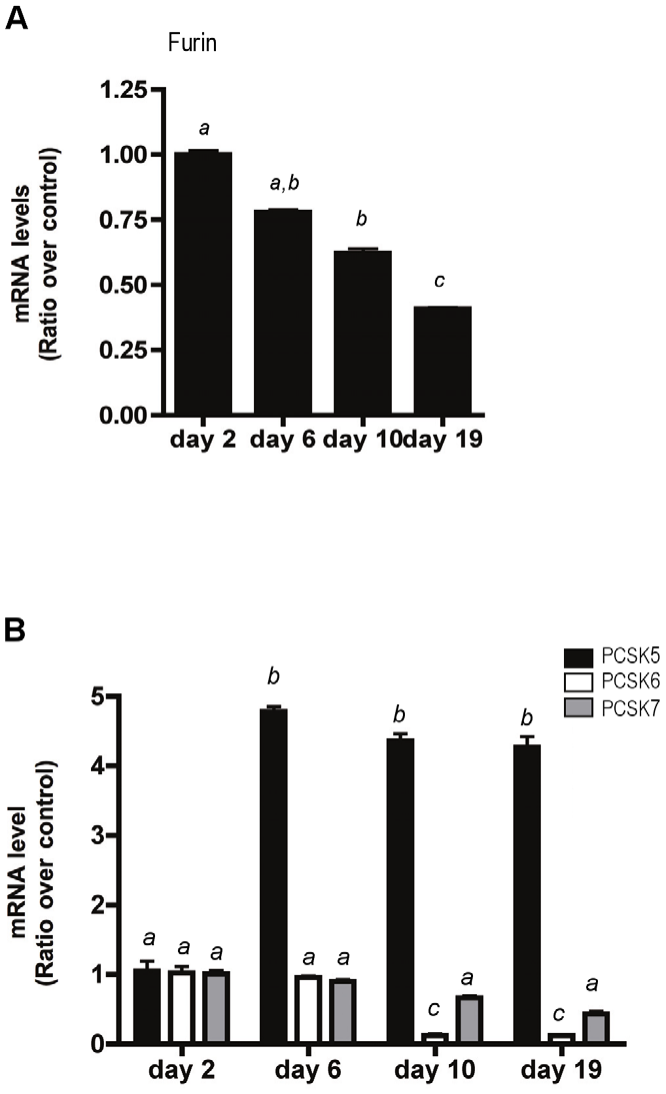
Expression of PCSKs in the postnatal mouse ovary. (A) Basal expression levels of furin mRNA in developing mouse ovaries was determined by real-time RT-PCR. The furin mRNA levels are shown as a ratio over the day 2 ovary mRNA levels (control). The bars labeled with different letters indicate statistically significant differences, as determined by Tukey's multiple comparison test (p<0.05). (B) Basal expression levels of PCSK5, PCSK6 and PCSK7 mRNA in developing mouse ovaries were determined by real-time RT-PCR and are shown as a ratio over day 2 ovary mRNA levels (control). The bars labeled with different letters indicate statistically significant differences, as determined by a two-way ANOVA followed by Bonferonni post-tests (p<0.05).

Our previous study demonstrated that activin treatment of pituitary gonadotrope (LβT2) cells positively regulated the production of furin mRNA through a pathway that involved the TGFβ receptor-activated transcription factors Smad2 and Smad3 [4]. With an increase of inhibin α- and β-subunit mRNA in growing follicles, we hypothesized an accompanying increase in furin mRNA levels over time. To test this, RNA was collected from ovaries of day 2, 6, 10 and 19 mice for quantitative real-time RT-PCR analysis. In contrast to our findings in the mouse pituitary, the relative levels of furin mRNA significantly decreased in the developing ovary when compared with day 2 ovaries (Figure 2A). The data suggests that the relative level of furin mRNA is inversely related to the level of the inhibin subunits in developing ovaries. We thus predicted that other enzymes belonging to the proconvertase family might be involved in inhibin subunit maturation. To determine the mRNA levels of PCSK5, PCSK6 and PCSK7 in whole ovaries, RNA was isolated from ovaries of d2, d6, d10, and d19 mice for quantitative real-time RT-PCR analysis. Of all enzymes examined, only PCSK5 mRNA was significantly elevated, by approximately 4-fold, when compared with day 2 ovaries (Figure 2B). In contrast, the relative amounts of PCSK6 and PCSK7 decreased when compared with day 2 ovaries, analogous to the mRNA expression profile of furin.

### Inhibin α-, β_A_- and β_B_-subunit mRNA transcript levels in isolated follicles

**Figure 3:**
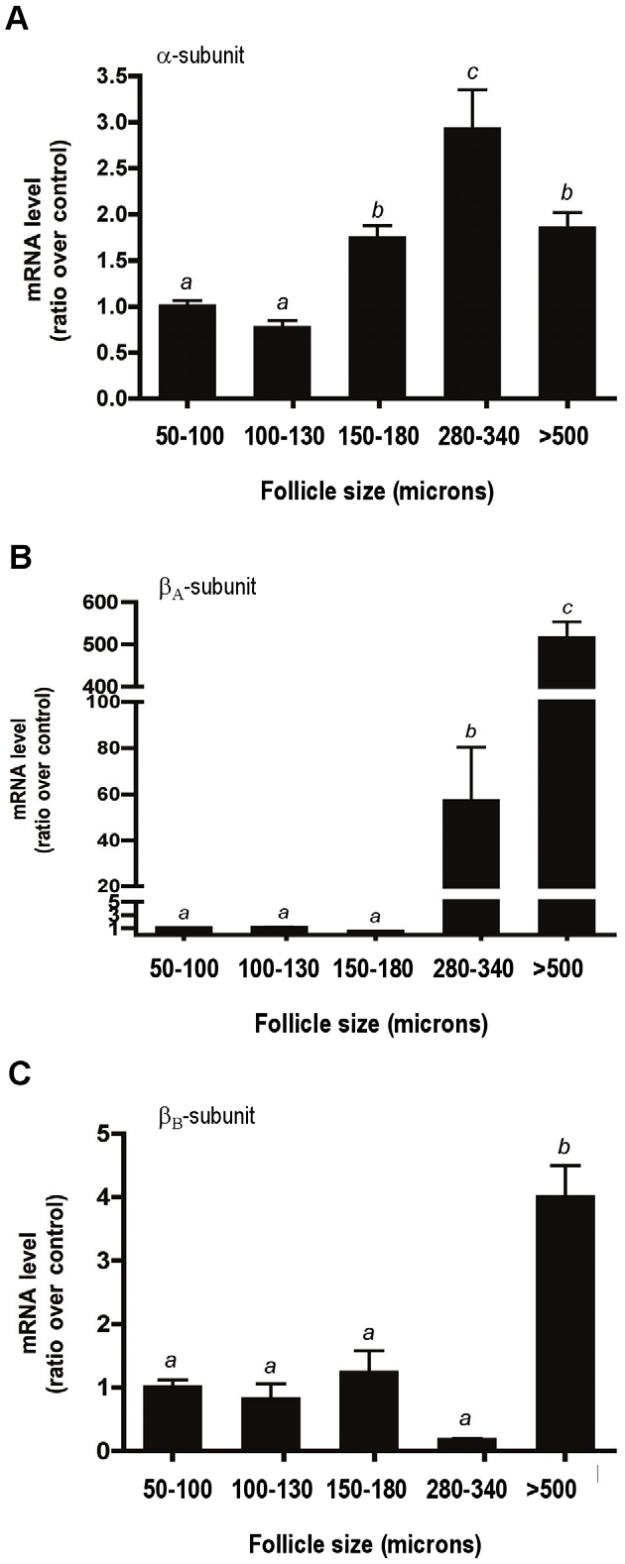
Expression levels of inhibin α-, β_A_- and β_B_-subunits in mechanically isolated mouse follicles. Follicles were dissected and grouped by size as described in the Materials and Methods. The mRNA levels of the α- (A), β_A_- (B) and β_B_- (C) subunits were measured using real-time RT-PCR and are shown as a ratio over the 50–100 micron follicle group levels (control). The bars labeled with different letters indicate statistically significant differences, as determined by Tukey's multiple comparison test (p<0.05).

Our goal was to determine which proconvertase enzymes cleave the inhibin subunits during ovary development and folliculogenesis. To identify follicle-specific inhibin subunit and proconvertase expression, we mechanically isolated follicles of various sizes from mouse ovaries. Follicles were isolated as previously described [21], [22] and RNA was collected from each group as described above in the Methods. Figure 3 graphically represents the mRNA expression of the inhibin α-, β_A_- and β_B_-subunits from each group of follicles. Compared with 50–100 micron follicles, the expression of the inhibin α-subunit increased approximately 1.7-fold in 150–180 micron size follicles (Figure 3A). The highest α-subunit mRNA levels (2.8 fold) was measured in 280–340 micron follicles. β_A_-subunit mRNA transcripts also increased significantly as follicles grew. The highest level of expression was observed in follicles greater than 500 microns (>500 fold) (Figure 3B). β_B_-subunit mRNA transcripts were also significantly higher in >500 micron follicles, with an approximate 4-fold increase in transcript levels compared to the smallest follicle group (Figure 3C). Thus, all three subunits are expressed and detectable in the appropriate follicle classes.

### Proconvertase mRNA transcript levels in isolated follicles

**Figure 4:**
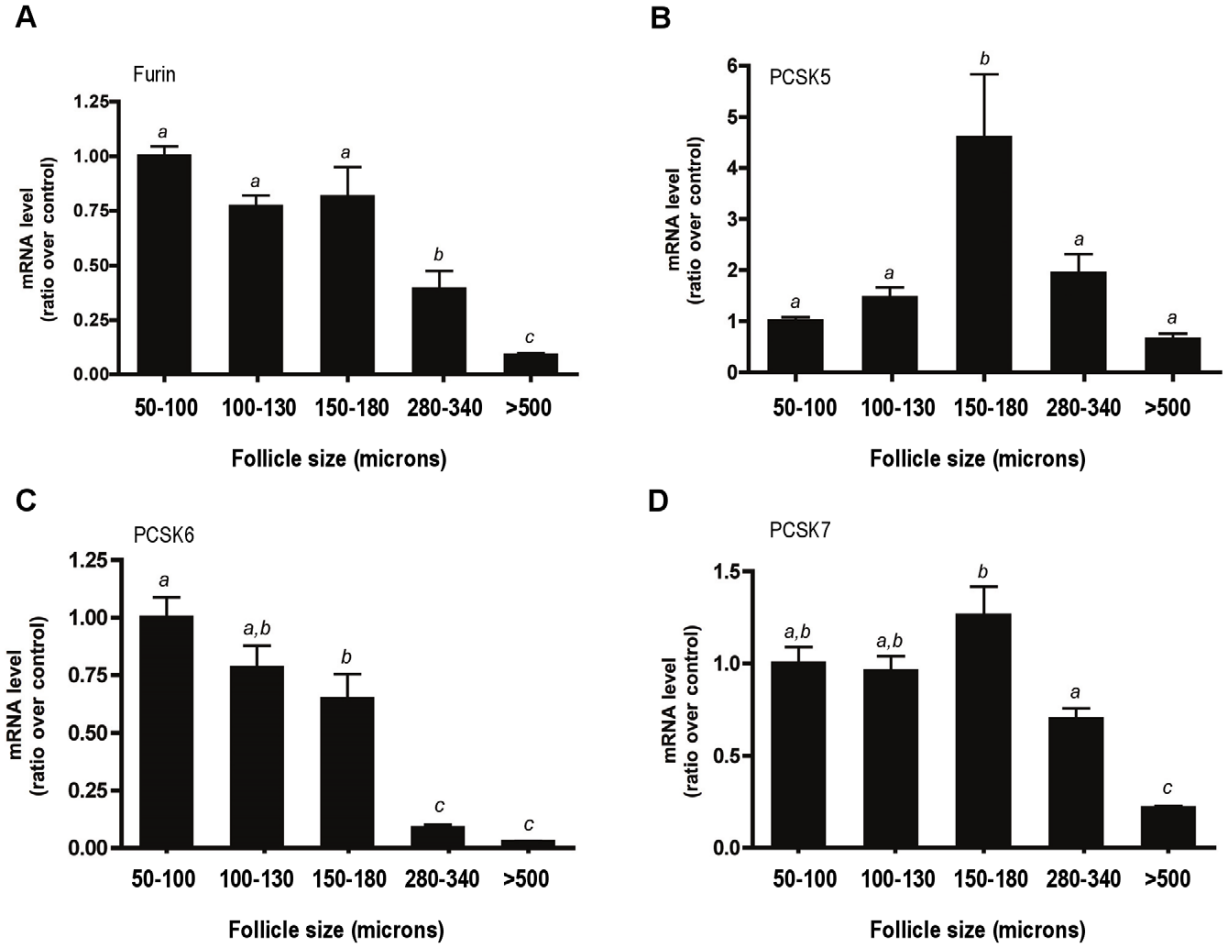
Expression levels of PCSKs in mechanically isolated mouse follicles. The mRNA levels of furin (A), PCSK5 (B), PCSK6 (C) and PCSK7 (D) subunits were measured using real-time RT-PCR and are shown as a ratio over the 50–100 micron follicle levels. The bars labeled with different letters indicate statistically significant differences, as determined by Tukey's multiple comparison test (p<0.05).

We next measured the mRNA levels of the proconvertases in each of the follicle classes (Figure 4). For each proconvertase examined, only the mRNA levels of PCSK5 increased significantly in 150–180 micron follicles (Figure 4B). This was consistent with the elevated PCSK5 mRNA levels that were observed in whole ovaries (Figure 2B). Also consistent with the data presented for the whole ovaries (Figure 2A), furin mRNA levels decreased significantly with follicle development. The trends observed for PCSK6 and PCSK7 expression were similar to that of furin, with a decrease in transcript levels as follicle size increased (Figures 2C, 4C and 4D). A concomitant increase in both inhibin α-subunit mRNA transcripts and PCSK5 mRNA transcripts in multilayer secondary follicles (150–180 microns) suggested that PC5/6 may be the proconvertase responsible for cleavage and activation of inhibin in the ovary.

### Activins regulate inhibin subunit and PCSK mRNA levels in secondary follicles

**Figure 5:**
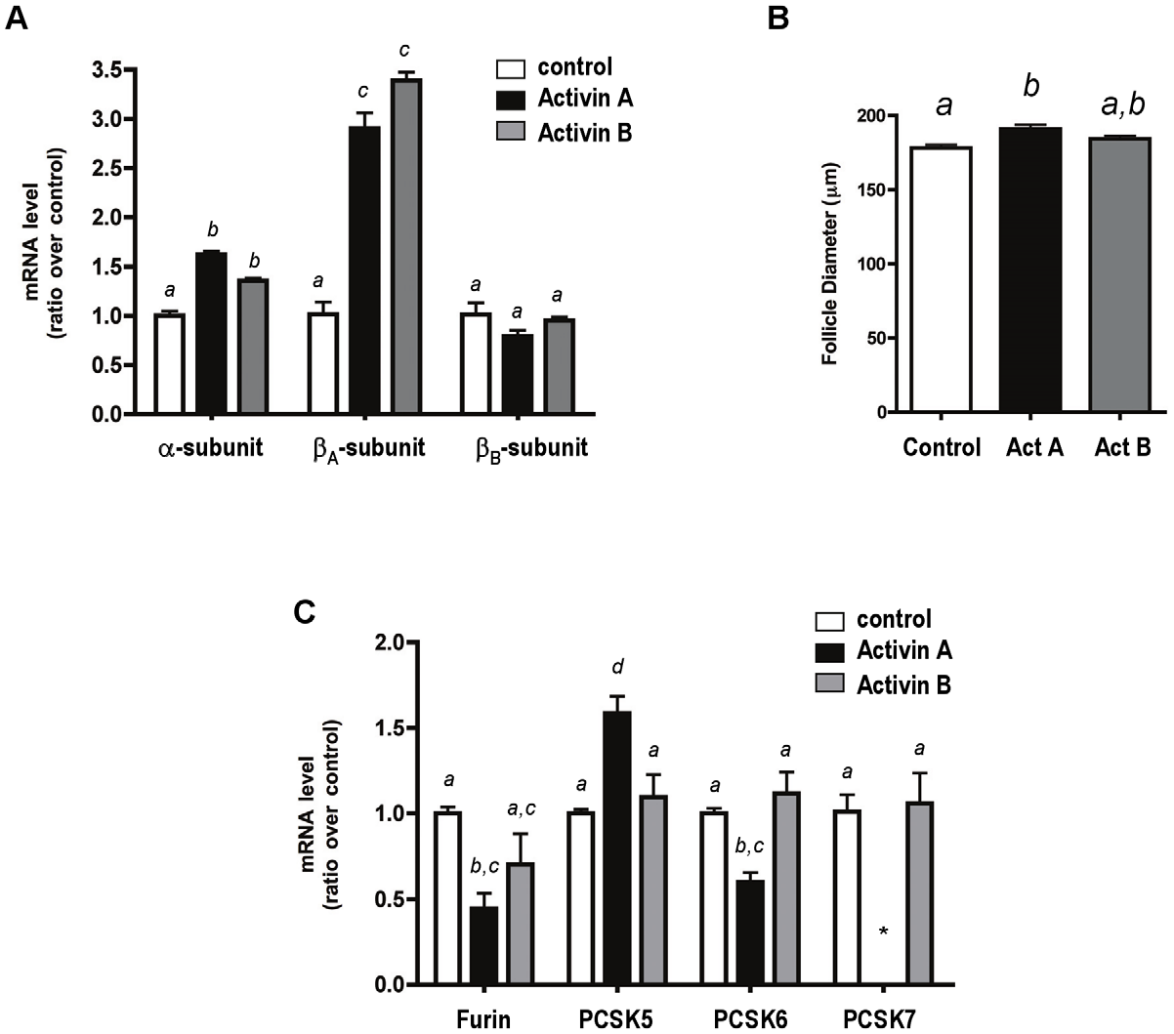
mRNA transcript levels of inhibin subunits and PCSKs in activin-treated *in vitro* cultured mouse follicles. Secondary follicles (100–130 microns) were mechanically isolated and cultured in alginate supplemented with activin A or activin B (50 ng/ml) for 4 days. (A) mRNA levels of the inhibin α-, β_A_- and β_B_-subunits. (B) Follicle diameter was measured in each treatment group after the 4-day culture period. A statistically significant difference in follicle diameter was noted in the presence of activin A, but not activin B. (C) mRNA levels of the PCSKs in each treatment group. Bars labeled with different letters indicate statistically significant differences, as determined by Tukey's multiple comparison test (p<0.05). *, PSCK7 mRNA transcripts could not be detected by real-time RT-PCR.

Since early follicle development is thought to be activin-dependent [27]; [28], [29], we questioned whether activins could effect the expression of inhibin subunits and PCSKs during follicle development. Two-layer secondary follicles (100–130 microns) were mechanically isolated from day 12 mouse ovaries and cultured for 4 days in the presence or absence of 50 ng/ml of either activin A or activin B [24], [25]. Two-layer secondary follicles were chosen in these experiments since no significant differences between any of the PCSK mRNA transcripts were observed at this stage (Figure 4). After this culture period, RNA was isolated from each group of follicles and the transcript levels of the inhibin subunits were assessed. Both the inhibin α-subunit and β_A_-subunit mRNA were positively affected by the presence of activin A or activin B (Figure 5A). A 1.5-fold increase was observed in the inhibin α-subunit expression with activin treatment. A 3-fold increase was observed for the β_A_-subunit transcript. The β_B_-subunit mRNA levels did not change in response to hormone treatment. Follicle diameters were measured for each group of treated follicles (Figure 5B). During this 4-day culture period, activin A-treated follicles grew significantly compared with controls [24], [25].

The mRNA transcript levels for each PCSK were then assessed in the activin-treated follicles. Compared with untreated follicles, furin and PCSK6 mRNA levels decreased significantly with activin A treatment, whereas PCSK5 mRNA levels increased significantly with activin A exposure. The observed changes were unique to activin A treatment, since little change in PCSK transcript levels was observed in follicles treated with activin B (Figure 5C). We also assessed if estrogen affected PCSK5 levels. No significant changes in PCSK5 expression occur following estrogen treatment. This finding is supported by Okada *et al*, (2005) which showed that the addition of estrogen to the culture medium had no significant effect on the levels of PCSK5 mRNA expression up to 6 days in culture. PCSK7 mRNA could not be detected in follicles treated with activin A. Taken together, the data provide evidence that activin A differentially affects expression levels of PCSK mRNA transcripts in secondary follicles cultured *in vitro*.

#### Relationship between PCSK gene expression and mature inhibin α-and β_B_-subunit levels

**Figure 6:**
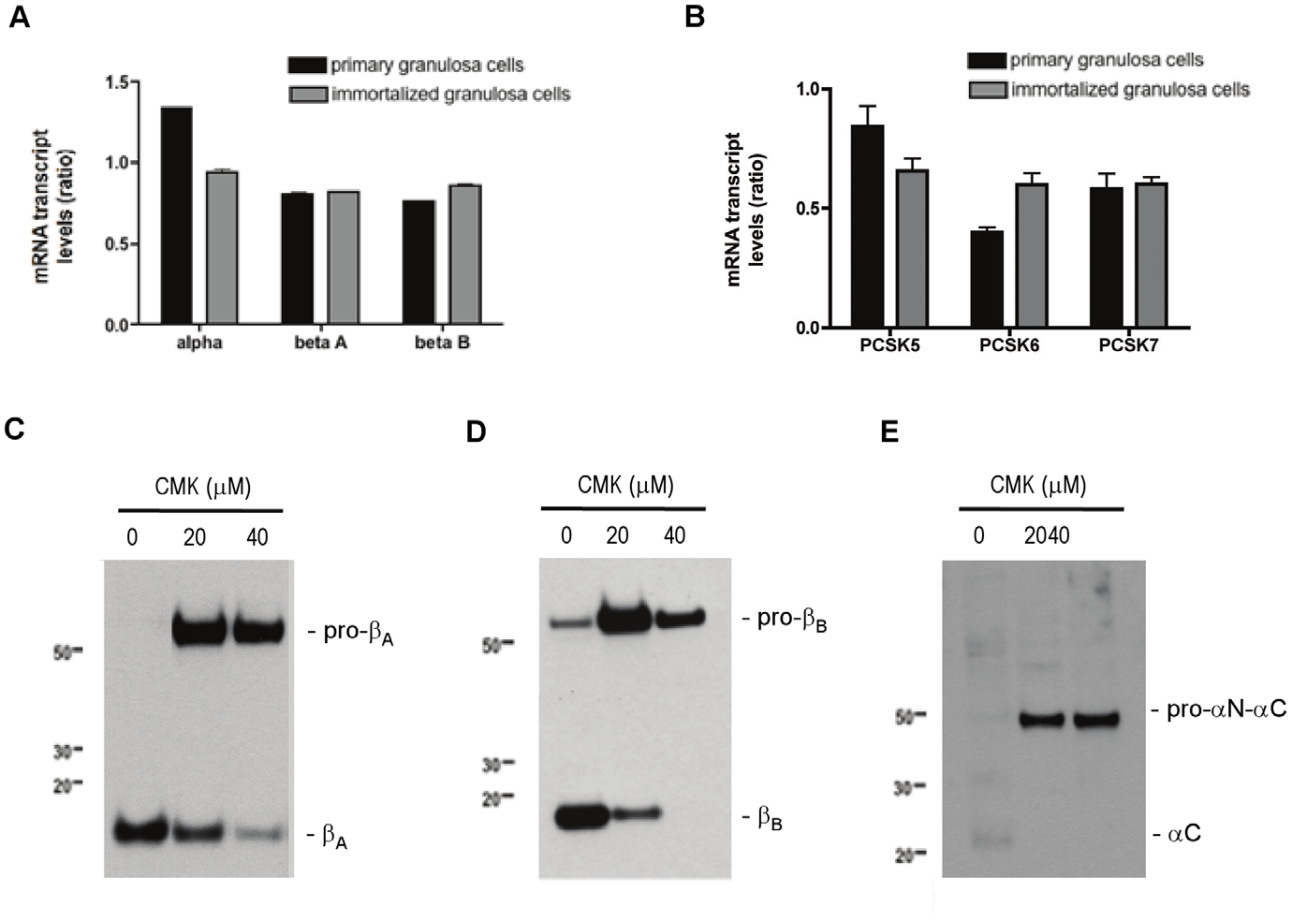
Expression levels of inhibin subunits and PCSKs in primary cultures of mouse granulosa cells and GRM02 cells. Following RNA extraction from both cell types, real time PCR analysis was performed using specific primers to the inhibin subunits (A) and the PCSK enzymes (B). Results are shown in the bar graph and are expressed as a ratio relative to the GAPDH control gene. Data are shown as mean ± SEM of three independent experiments. (C) Proconvertase enzymes are necessary for processing of inhibin subunits. GRMO2 cells were cultured for 48 h in the presence of the proconvertase inhibitor dec-RVRK-CMK (CMK). TCA-precipitated media was separated by SDS-PAGE under reducing conditions and imunoblotted using an anti-inhibin β_A_-subunit antibody, (D) anti-inhibin β_B_-subunit antibody or (E) anti-inhibin α-subunit antibody. The results are representative of three independent experiments.

The expression of the inhibin subunits and PCSK enzymes were quantitatively assessed by real time PCR of total RNA isolated from a primary culture of adult mouse granulosa cells and immortalized mouse granulosa (GRMO2) cells. In our characterization of these two cultures of granulosa cells, real time PCR data showed that the basal mRNA levels of the inhibin α-, β_A_-and β_B_-subunits were comparable in both culture conditions (Figure 6A). A similar distribution pattern of PCSK5, PCSK6 and PCSK7 mRNA transcripts were also detected in both sources of granulosa cells (Figure 6B). We next investigated the relationship between PCSKs and inhibin α-, β_A_-and β_B_-subunit processing using GRMO2 cells. Cells were treated with a potent and irreversible inhibitor of proconvertase enzymes, decanoyl-RVKR-CMK (CMK), for 1 hour. Media was collected, TCA-precipitated and subject to immunoblot analysis. As shown in Figures 6C–6E, processing of each of the inhibin subunits was inhibited by the presence of CMK. Accumulation of unprocessed inhibin α-subunit in the media suggested that this inhibitor affects cleavage but not secretion. The data imply a direct relationship between proconvertase enzyme activity and the maturation of inhibin α-, β_A_-and β_B_-subunits expressed in granulosa cells.

**Figure 7:**
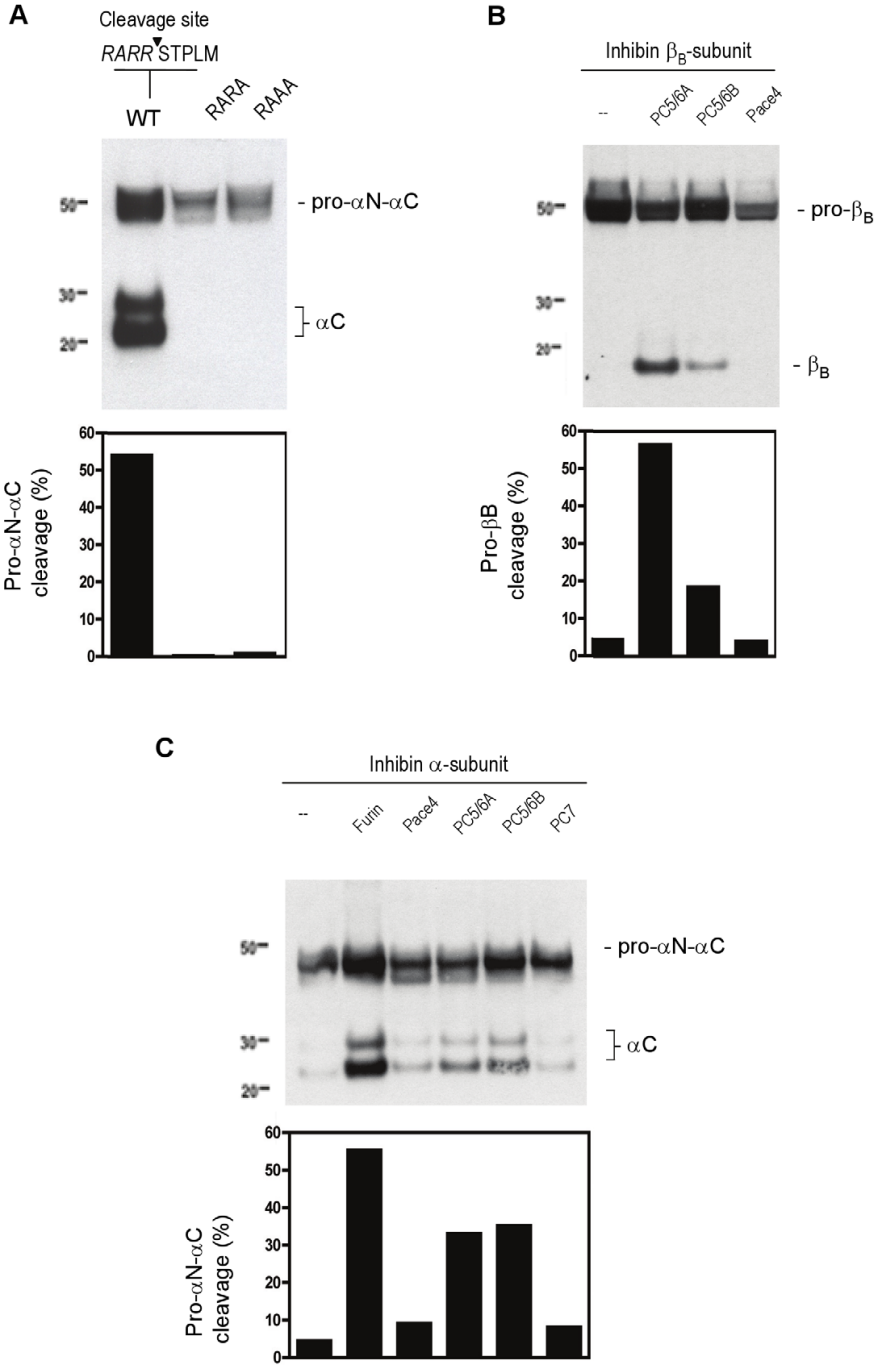
Role of PCSKs in inhibin subunit cleavage. (A) Expression of the wild type inhibin α-subunit (RARR) or the α-subunit with modified cleavage recognition sites (RARA and RAAA) in HEK 293 cells. Culture media were examined by immunoblot analysis under reducing conditions using an anti-inhibin α-subunit antibody. Processing of the inhibin β_B_-subunit (B) and inhibin α-subunit (C) by proconvertases. LoVo cells were transiently transfected with expression plasmids for furin, PC5/6A, PC5/6B, Pace4 and PC7. Media from cells was TCA-precipitated, separated by SDS-PAGE under reducing conditions and examined by immunoblot analysis. The corresponding % of band intensities were deduced from the ratio of (β_B_)/(pro-β_B_+β_B_) for the β_B_-subunit; or (αC)/(pro-αN-αC+αC) for the α-subunit. Results are representative of two independent experiments.

We further investigated the potential role of the C-terminal proteolytic processing in the secretion of the inhibin α-subunit. We previously reported the importance of this processing site in the inhibin β_B_ -subunit [4]. The predicted cleavage motif (RARR) at amino acids 229–232 of the inhibin α-subunit (pro-αN-αC) was verified by site-directed mutagenesis experiments. Two inhibin α-subunit mutants (RARA and RAAA) were generated and expressed in HEK293 cells. Compared with controls, cells containing either cleavage site mutant inhibin α-subunit produced unprocessed inhibin (confirming the earlier observation that unprocessed inhibin can be secreted), but undetectable levels of secreted mature inhibin (αC) subunit (Figure 7A). These results confirmed that cleavage of the inhibin α-subunit occurs at the consensus cleavage motif and that mutagenesis of only the last arginine (the RARA mutant) is sufficient to abolish C-terminal proteolytic processing. The two arginine amino acids are not equally important for processing as previously described [30] since the secretion profile of this mutant (RAAA) did not differ significantly from the RARA mutant.

To further validate that the inhibin α- and β-subunits are targets of proconvertase processing, the furin-deficient (LoVo) cell line was utilized. LoVo cells were transiently transfected with the inhibin β_B_- or α-subunit and expression vectors for PC5/6A, PC5/6B, Pace 4 or PC7. After 48 h, the media was collected, TCA precipitated and subject to immunoblotting procedures under reducing conditions. Overexpression of PC5/6A or PC5/6B resulted in the cleavage and secretion of pro-β_B_ proteins, and PC5/6A was more efficient than PC5/6B (Figure 7B). Overexpression of Pace4 or PC7 (data not shown) did not cleave pro-β_B_ protein. Consistent with our previous report, furin cleaved inhibin pro-αN-αC (Figure 7C) [4]. Overexpression of either PC5/6A or PC5/6B cleaved the inhibin precursor and more mature αC protein was observed when compared with control. Very low levels of αC were also evident with Pace4 and PC7 overexpression, suggesting that these proconvertase enzymes are able to cleave the inhibin α-subunit with different efficiencies. To our knowledge, the data presented here provide the first evidence that the inhibin β_B_- and α-subunits are targets of PC5/6 cleavage.

## Discussion

Our findings represent an extension of previous work from our group suggesting that proconvertases mediate processing of inhibin subunits in the pituitary [4]. While we found previously that furin cleaves the α- and β_B_-subunits in pituitary gonadotrope cells, here, we observed a decrease in the expression of furin mRNA transcripts during follicle development. This finding is consistent with a previous report examining furin levels in the ovary of the medaka, *Oryzias latipes* [31]. Ogiwara *et al* (2004) demonstrated the greatest abundance of furin mRNA transcripts in small follicles when compared to medium and large sized follicles. Our findings suggest that other proconvertase pathways are involved in the processing of inhibin in the ovary. Our work has produced new evidence that PCSK5, and primarily the PC5/6A isoform, cleaves the inhibin subunits. We demonstrate that the expression of the inhibin subunits increases in whole ovaries and mechanically isolated follicles, and that the precursor proteins are proteolytically activated by PCSK5 (encoding PC5/6 protein) that is also upregulated during follicle development.

Inhibin A and inhibin B are present in the circulation at very distinct times during the female reproductive cycle [32], [33], [34], [35], [36], and local activin levels within the ovary also change during folliculogenesis in a highly regulated manner [35]. As all inhibin subunits are expressed in developing follicles, the question becomes how processing, assembly and secretion of mature inhibin and activin dimers are regulated to achieve cyclical, discordant serum hormone levels. We demonstrate that the spatial and temporal expression of the proconvertase PCSK5 overlaps with the expression and processing of mature inhibin subunits in the ovary during follicle development. This suggests that the proconvertase PC5/6 may be involved in inhibin A subunit processing in small follicles, as PCSK5 expression increases during the transition from a 2-layer secondary follicle (100–130 microns) to a preantral follicle (150–180 microns). While our work has shown that the inhibin α-subunit is cleaved by furin (pituitary) and PC5/6 (ovary), it is important to note that cleavage is not required for inhibin dimerization or secretion. Thus, other mechanisms, which are likely dependent on the β_A_- and β_B_-subunits, must be in place in order to achieve the discordant levels of inhibin A and inhibin B during the reproductive cycle.

Activin A is known to be a local regulator of early follicle development [27], [28], [29]. We show that activin A, but not activin B, positively regulates PCSK5 mRNA levels in cultured follicles. The regulation of proconvertase enzymes by hormones is supported by a study in prepubertal rats, which demonstrates gonadotrophin regulation of PCSK5 mRNA [37]. In our proposed model, local activin A acts in an autocrine/paracrine manner to stimulate processing of mature inhibin subunits in granulosa cells. Activin B had no effect on the expression of any of the PCSKs studied. Endogenous expression of the activin β_B_-subunit in the ovary is relatively low compared with the activin β_A_-subunit; therefore, it is likely that activin A plays the more prominent role in follicle development by regulating inhibin subunit processing during the transition from the secondary follicle stage. It is possible that follicles cultured to the antral stage, when β_B_-subunit expression begins to increase, might have demonstrated activin B-stimulated PCSK5 expression. The effect of inhibins on PCSK5 expression and activity will be investigated in future studies.

It is also interesting that activin A negatively regulates PCSK6 and furin (and possibly PCSK7). This finding confirms the observations of Diaz *et al* (2008), who showed suppression of granulosa cell PCSK6 expression by oocytes [38]. They suggested that PCSK6 inhibition might represent a mechanism for regulating the activity of the TGF beta superfamily members within the developing follicle. There are numerous examples of paracrine signaling that occur between and among the various cells that make up the follicle, particularly during early follicle development [1]. Inhibin subunit processing may be regulated by an activin feedback loop that occurs between granulosa cells to ensure that PC5/6 mediates inhibin A processing proceeds through the preantral stage, followed by processing of inhibin B in larger follicles. The data suggests that the concurrent increase in inhibin subunit processing and PSCK5 expression are important mediators of follicle development. Nonetheless, it is unclear why activin treatment decreases furin mRNA transcripts in our follicle culture experiments. Transcriptional regulation studies using a reporter construct with the furin promoter transiently transfected into murine granulosa cells and treated with activin is unresponsive. It appears that downregulation of furin mRNA may be indirect and Smad-independent (data not shown). Whether the oocyte secretes factors that regulate furin mRNA levels in response to activin warrants further investigation.

In summary, we have shown that the expression of PCSK5 (encoding PC5/6) correlates with the expression of the inhibin subunits in the mouse ovary and in individual follicles. Expression of PCSK5 peaks during the transition from secondary to preantral follicle stages, and is upregulated by activin A treatment of cultured mouse follicles. PC5/6 was shown to process the α- and β_B_-subunits in cultured cells lines. Future work will continue to focus on how inhibin subunit processing is coordinated with subunit expression, dimerization and secretion to achieve the cyclical patterns of ovarian inhibin and activin production and activity during the reproductive cycle.
